# Efforts and Support Needs of Japanese Nurses Working Alongside Migrant Nurses in Japan: A Qualitative Study

**DOI:** 10.3390/nursrep16070241

**Published:** 2026-07-13

**Authors:** Rina Shoki, Anna Kono, Edward Barroga, Yasuko Nagamatsu

**Affiliations:** 1 Graduate School of Nursing Science, St. Luke’s International University, 10-1 Akashi-cho, Chuo-ku, Tokyo 104-0044, Japan; edward-barroga@slcn.ac.jp (E.B.); sarah-nagamatsu@slcn.ac.jp (Y.N.); 2 Faculty of Nursing at Saitama, Japanese Red Cross College of Nursing, 8-7-19 Kamiochiai, Chuo-ku, Saitama 338-0001, Japan; 3 Department of Nursing, Faculty of Health Care, Teikyo Heisei University, 4-21-2 Nakano, Nakano-ku, Tokyo 164-8530, Japan; a.kono@thu.ac.jp; 4 Medical English Education Center, School of Medicine, Showa Medical University, 1-19-14 Hatanodai, Shinagawa-ku, Tokyo 142-8777, Japan

**Keywords:** migrant nurses, nurse migration, international nursing, transcultural nursing, nursing practice, education, support, qualitative study

## Abstract

**Background/Objectives:** The employment of migrant nurses requires structured support from local nursing staff. However, multiple challenges persist in facilitating effective collaboration. We aimed to clarify the efforts made by Japanese nurses and identify their support needs when working alongside migrant nurses in Japan. **Methods:** We adopted a qualitative descriptive design. Eighteen Japanese nurses with experience working alongside migrant nurses were recruited through hospitals employing migrant nurses. Semistructured interviews were conducted, and data were analyzed using qualitative descriptive analysis. We classified the codes derived from the participants’ narratives and inductively generated categories. **Results:** Japanese nurses’ efforts in working alongside migrant nurses were classified into five categories: “Overcoming language barriers,” “Respecting the nursing practices and cultural backgrounds of migrant nurses,” “Encouraging migrant nurses to gain self-confidence,” “Engaging Japanese staff in establishing support systems for migrant nurses,” and “Addressing the personal challenges faced by migrant nurses working abroad.” Three categories of support needs were identified: “Learning about nursing practices and cultural backgrounds of migrant nurses,” “Information exchange on how to support migrant nurses,” and “Support systems for enhancing migrant nurses’ competencies in Japan.” **Conclusions:** Japanese nurses demonstrated active efforts to address differences in language, culture, and nursing practices, while collaboratively establishing support systems involving other staff members. They also extended support to aspects of migrant nurses’ personal lives. However, additional institutional and external support is required. Structured opportunities should be developed to enhance their understanding of migrant nurses’ backgrounds, alongside continuing education initiatives to support migrant nurses’ integration into the Japanese healthcare system.

## 1. Introduction

Japan has accepted migrant nurses from Indonesia, the Philippines, and Vietnam under the Economic Partnership Agreement (EPA) [1]. Under this system, migrant nurses who hold nursing qualifications and have several years of clinical experience in their home countries are accepted into Japanese hospitals as “EPA nurse candidates” [2]. While preparing for the Japanese national nursing licensure examination, these candidates are allowed to work as nursing assistants in clinical settings [2]. By 2025, 1788 individuals had entered Japan as EPA nurse candidates, of whom 719 passed the national nursing examination [2]. Those who obtain licensure are expected to continue working in Japan for extended periods, as employment restrictions are lifted [2]. However, the turnover rate among migrant nurses remains high [3].

Migrant nurses working in Japan experience difficulties related to the Japanese language, cultural differences, and variations in nursing practices [4]. Studies conducted outside Japan have also identified these issues as major challenges faced by migrant nurses during adaptation to their host countries [5]. These challenges may limit their ability to perform nursing tasks independently compared with newly graduated Japanese nurses, and they are associated with increased workload burden and psychological stress [6,7]. Furthermore, in addition to the EPA program, the number of Chinese nurses accepted in Japan has increased since around 2010 [8]. As Chinese nurses are recruited through private organizations, the exact number of those accepted remains unclear [8]. Nevertheless, they appear to encounter similar challenges in adapting to the Japanese healthcare environment [9]. Therefore, to support the retention and effective integration of migrant nurses, it is essential that Japanese nurses provide structured and context-specific support tailored to individual competencies [10].

On the other hand, Japanese nursing administrators and educators in hospitals employing migrant nurses face challenges in communication during clinical practice, accommodating religious beliefs, and providing instruction on nursing skills that may differ from those in the nurses’ home countries [11]. Although the employment of migrant nurses may inspire Japanese nurses and broaden their perspectives, it may also present challenges, including restrictions on the duties migrant nurses can perform and increased staff workload associated with training [12]. They also report psychological stress related to compensating for workload imbalances, delivering real-time supervision, and managing unexpected staff turnover [11]. These challenges stem from the lack of structured systems to support migrant nurses’ integration into Japanese hospitals and the limited availability of preparatory educational resources for both migrant nurses and Japanese nurses. From a human resource management perspective, nursing managers support migrant nurses’ career development by offering opportunities for Japanese language learning alongside existing programs for Japanese nurses [13]. Furthermore, there is a need to foster an organizational culture in which migrant and Japanese nurses can mutually respect cultural differences and collaborate effectively [13].

Previous studies have identified challenges in supporting migrant nurses from the perspectives of administrators and educators. However, migrant nurses continue to experience difficulties with information-dense communication, even after passing the Japanese national nursing examination [14]. Consequently, Japanese colleagues play a crucial role in supporting migrant nurses in their day-to-day work. However, the specific strategies Japanese nurses use in their daily practice, as well as their support needs that arise from these experiences, remain insufficiently understood.

In this study, we aimed to identify the efforts made by Japanese nurses to work alongside migrant nurses in Japan, as well as their support needs. The findings are anticipated to contribute to the development of practical support strategies and inform organizational approaches in order to promote effective collaboration and sustainable workforce integration within the Japanese healthcare system.

## 2. Materials and Methods

### 2.1. Study Design

This study employed a qualitative descriptive design using semistructured interviews to explore Japanese nurses’ experiences and support needs when working with migrant nurses.

### 2.2. Definition of Terms

In this study, “migrant nurses” are defined as individuals whose native language is not Japanese and who received nursing education outside Japan, subsequently passed the Japanese national nursing licensure examination, and are currently employed at Japanese hospitals.

“Support needs” refers to the types of support that Japanese nurses perceive as necessary at the individual or organizational level to promote effective collaboration with migrant nurses. This concept is used broadly to encompass educational, informational, emotional, and organizational support.

### 2.3. Participants

Participants were Japanese nurses whose first language was Japanese and who had experience working with migrant nurses in clinical settings in Japan. We requested cooperation for the study from the nursing directors at 145 hospitals that currently or previously employed migrant nurses and recruited participants from the 33 hospitals that agreed to participate in the study. In addition, snowball sampling was used to recruit additional participants and ensure adequate sample size and diversity of experiences.

### 2.4. Data Collection

#### 2.4.1. Participant Characteristics

Data on participants’ demographic and professional characteristics were collected, including years of nursing experience, roles in supporting migrant nurses, the number and countries of origin of migrant nurses they have worked with, and the duration of their experience working with migrant nurses.

#### 2.4.2. Interviews

Semistructured interviews were conducted using an interview guide developed specifically for this study through discussions among the authors. During the interviews, participants were asked to describe their experiences of working with migrant nurses after they had obtained nursing licensure. The key questions were as follows:

1. Please tell me about any migrant nurses you currently work with or have worked with in the past.
2. Please describe how you have interacted with migrant nurses, either currently or in the past.
3. What challenges do you face when working with migrant nurses?
4. What efforts have you made to address these challenges when working alongside migrant nurses?
5. What types of support would be helpful for you and your Japanese colleagues to facilitate collaboration with migrant nurses?

Interviews were conducted in Japanese. Because participants resided in various regions across Japan, all interviews were conducted online. With the participants’ consent, the interviews were audio-recorded, supplemented with field notes, and transcribed verbatim for analysis. All interviews were conducted by the first author, who has experience in qualitative interviewing.

### 2.5. Data Analysis

Data were analyzed using a qualitative descriptive approach based on the method described by Gregg et al. [15]. All interview data were transcribed verbatim. Through open coding, text segments relevant to the research topic were identified and organized into meaning units using participants’ original expressions, after which the meaning units were coded. The codes were compared for similarities and differences, and subcategories were inductively generated based on shared meanings. These subcategories were further refined and abstracted into broader categories through an iterative process. Coding and categorization processes were primarily performed by a single author and conducted manually without the use of data analysis software.

To ensure analytical rigor, multiple strategies were employed. Credibility was enhanced through member checking with one participant, who reviewed the analysis and confirmed that the findings accurately reflected their experiences. The coding process and category development were continuously reviewed and discussed among three researchers to achieve consensus. In addition, a co-researcher with extensive experience in qualitative research provided supervision throughout the analysis process. Dependability and confirmability were strengthened by maintaining consistency in the analytic procedure, documenting coding and category development, and discussing interpretive differences among the researchers until consensus was reached. The research team also engaged in reflexive discussion regarding how their professional backgrounds and assumptions might influence the interpretation of the data. Based on Gregg et al.’s sampling method [15], data saturation was determined to have been reached when no new categories emerged during the analysis process. Transferability was considered by describing the participants and study context; however, because this study was based on Japanese nurses’ perspectives, the findings may not be directly transferable to all clinical settings or to migrant nurses’ own experiences. The interviews were conducted and analyzed in Japanese. The quotations were first translated using ChatGPT Plus (GPT-5.2) and subsequently reviewed by a professional English editor. Three authors then checked the translations against the original Japanese transcripts to ensure that the intended meanings and nuances were accurately preserved. The interpretation and categorization of the data were performed by the research team and were not based on the automated translation output.

### 2.6. Study Period

This study was conducted from May 2022 to November 2022.

### 2.7. Ethical Considerations

This study was conducted with the approval of the Ethics Review Committee of St. Luke’s International University (Approval Number: 21-A098). Participants were provided with written explanations regarding the purpose and methods of the study, the voluntary nature of participation and the option to withdraw at any time, the protection of personal information, and the potential benefits and drawbacks of participating in this research. Written informed consent was obtained from all participants prior to data collection.

## 3. Results

### 3.1. Characteristics of Study Participants (Table 1)

Eighteen Japanese nurses participated in the study. Their mean years of nursing experience was 18.3 years. Each participant was interviewed once. The interview durations ranged from 26 to 70 min, with an average duration of 47 min.

### 3.2. Results of the Interviews

#### 3.2.1. Efforts Made by Japanese Nurses to Work Alongside Migrant Nurses (Table 2)

The efforts made by Japanese nurses to work alongside migrant nurses were classified into five categories: “Overcoming language barriers,” “Respecting the nursing practices and cultural backgrounds of migrant nurses,” “Encouraging migrant nurses to gain self-confidence,” “Engaging Japanese staff in establishing support systems for migrant nurses,” and “Addressing the personal challenges faced by migrant nurses working abroad”.

Subcategories are indicated by [ ] and codes by < >. Illustrative quotations are presented in italics with participant IDs, and any necessary supplementary information is provided in parentheses ( ).

The results presented below reflect the participants’ perceptions and experiences of working alongside migrant nurses. These findings should not be interpreted as objective characteristics of migrant nurses or as generalizable to any specific national group.

- Overcoming Language Barriers

This category comprised five subcategories and 18 codes as follows: [Using clear and simplified language to facilitate understanding among migrant nurses], [Providing repeated and sustained explanations until understanding is achieved], [Supporting tasks requiring advanced Japanese language proficiency], [Developing and utilizing tools to overcome language barriers], and [Providing continuous opportunities for migrant nurses to learn and improve Japanese language skills].

Because communication styles typically used among Japanese nurses did not always ensure accurate understanding by migrant nurses, participants used <simplified and textbook-based sentence patterns> and <repeatedly explained patient safety–critical issues until understanding was confirmed>. In addition, when Japanese staff had difficulty in understanding information provided by migrant nurses during handovers, participants supported communication by <clarifying handover information>.

*“(When speaking with migrant nurses,) I try to use textbook-style expressions. For example, saying something like ‘That’s not good’ isn’t understood at all, so I instead say things like ‘You shouldn’t do that’ or ‘That is not allowed.’ Speaking in that way makes it easier for them to understand.”*

(002)

*“If I could anticipate even the slightest possibility that something might lead to an incident or accident, I felt it was necessary to speak up. In such cases, I would take the time to explain patiently and persistently. Even if they seemed reluctant, I would say, ‘No, that’s not quite right—please listen once more,’ and I would repeat the explanation as many times as needed.”*

(003)

*“When a Japanese nurse was unable to fully understand the information provided in a handover by a migrant nurse, I ask the migrant nurse, ‘do you mean this?’ and support the handover by helping to clarify the information.”*

(015)

For education, participants <developed illustrated manuals to facilitate visual understanding> and <provided feedback on written Japanese used in nursing records to support language development>.

*“We found that the materials previously used for new graduated Japanese nurses were not suitable for educating migrant nurses. For procedures that were difficult to visualize, we consulted with migrant nurses to explore how they could best learn them, and revised the educational materials by incorporating illustrations and photographs to facilitate understanding.”*

(005)

*“Some people may have thought that it was sufficient as long as the Japanese was understood to some extent; however, she (the migrant nurse) wanted to continue improving, and we agreed that it would be better to write in clear and well-structured Japanese. To help her express herself more effectively in Japanese, I corrected the Japanese used in the nursing records she had written.”*

(015)

- Respecting the Nursing Practices and Cultural Backgrounds of Migrant Nurses

This category comprised three subcategories and eight codes: [Teaching Japanese nursing practices while acknowledging perspectives from migrant nurses], [Adapting instructional approaches to be culturally appropriate], and [Creating work environments that accommodate religious practices]. This category focuses on professional interactions within the workplace and reflects Japanese nurses’ efforts to support migrant nurses while acknowledging and respecting their values.

Participants balanced instruction with cultural sensitivity. When migrant nurses were confused by differences between nursing care in Japan and care practices in their home countries, participants <explained the purpose and significance of Japanese nursing practices to support their understanding>. Recognizing that migrant nurses had professional experience in their home countries, participants <sought to acknowledge their perspectives before providing corrective guidance>. They also <adapted workloads to accommodate religious practices>, such as reducing physically demanding tasks during Ramadan.

*“A migrant nurse said that tasks such as elimination care were not considered part of nurses’ work in her home country, as they were performed by family members. She appeared confused by the discrepancy between nursing practices in her home country and those in Japan, so I repeatedly explained, ‘In Japanese nursing, these aspects are valued. Providing emotional support and helping patients prepare to return home are also important parts,’ and gradually helped her internalize this understanding.”*

(013)

*“Some migrant nurses often showed a tendency not to accept instructions unless they aligned with their own decisions. I thought this might be influenced by their cultural background, so rather than correcting them from a position of authority by saying, ‘That’s not right,’ I first listened to and acknowledged their perspectives.”*

(001)

*“During Ramadan, as they (Muslim nurses) are not able to drink water at all, we try to avoid assigning physically demanding tasks, such as assisting with bathing that induces sweating, during the daytime.”*

(008)

- Encouraging Migrant Nurses to Gain Self-Confidence

This category comprised two subcategories and five codes: [Recognizing migrant nurses as qualified professionals in their home countries] and [Assigning tasks that leverage migrant nurses’ individual strengths].

Participants understood that migrant nurses might feel frustrated when language and other barriers limited their ability to work independently. To support their confidence, participants recognized migrant nurses’ prior competencies by <allowing performance of familiar clinical skills independently> and <assigning roles that drew on their unique experiences>, such as supporting communication for other foreign trainees.

*“He (the migrant nurse) was already able to assist directly in surgery as a fully contributing member. I thought that if we helped him become independent in certain areas as early as possible and encouraged other staff (Japanese nurses) to rely on him, it might reduce dissatisfaction on both sides.”*

(009)

*“I don’t think there are many people who have worked this long under the EPA program, so I would like her to share her experiences. Our hospital also accepts technical intern trainees from Vietnam, and we have assigned her (a migrant nurse) a role in supporting those trainees.”*

(015)

- Engaging Japanese Staff in Establishing Support Systems for Migrant Nurses

This category comprised two subcategories and five codes: [Raising awareness among Japanese staff regarding the characteristics and needs of migrant nurses] and [Acting as intermediaries between migrant nurses and Japanese staff]. Unlike the previous category, which focuses on interpersonal interactions, this category reflects efforts to develop collaborative organizational support systems that facilitate migrant nurses’ adaption.

Participants occasionally <spoke on behalf of migrant nurses to convey their thoughts that Japanese nurses may not have recognized>, and <mediated misunderstandings between migrant nurses and Japanese staff>.

*“During Ramadan, we adjusted break times during night shifts so that migrant nurses could have breakfast before sunrise. Anticipating that this might lead to dissatisfaction among Japanese staff, we provided detailed explanations about Ramadan, emphasizing the importance of managing one’s physical condition and the need for support over the approximately month-long period, thereby enabling staff to support migrant nurses.”*

(013)

*“For example, there were many instances in which migrant nurses’ thoughts and intentions were not conveyed to Japanese nursing assistants, or their explanations to physicians were insufficient and not fully understood. In such cases, I would step in to listen to both sides and help clarify and resolve the situation. I made sure not to let things end with one-sided assumptions, such as Japanese nurses saying, ‘I told that person (the migrant nurse), but they didn’t do it.”*

(015)

- Addressing the Personal Challenges Faced by Migrant Nurses Working Abroad

This category comprised three subcategories and eight codes: [Responding to migrant nurses’ needs related to returning to their home countries], [Providing support for challenges encountered in daily life in Japan], and [Supporting the needs of accompanying family members]. This category extends beyond workplace-based support and captures efforts to address the personal and everyday life challenges encountered by migrant nurses living and working abroad.

Participants <arranged extended leave to allow migrant nurses to return to their home countries> and <considered long-term life plans in employment discussions>. They also provided support for daily living issues, such as communication through messaging applications and assistance with essential needs.

*“(Regarding long-term leave for returning home,) we try to provide about one month of consecutive leave by combining it with regular days off. In cases such as marriage, some may wish to return (to their home countries) for up to two months. Although we cannot offer such extended leave every year, we generally provide it about once every two years.”*

(008)

*“As their life plans differ from those of Japanese nurses, working in Japan is incorporated into their broader life trajectories. They may therefore get married or have children unexpectedly. For this reason, we communicate with them so that their perspectives on the timing of returning to their home countries can be incorporated into their employment plans.”*

(010)

In the subcategory [Providing support for challenges encountered in daily life in Japan], participants <exchanged contact information with migrant nurses to provide support for daily life issues> and also <prepared essential daily necessities>. These findings indicate that support extended beyond the workplace into broader social and family contexts.

*“In our private time, we stay connected with migrant nurses* via *LINE. They often encounter various difficulties in their daily lives—for example, receiving notices about unpaid electricity bills or experiencing internet connectivity problems. They report such issues* via *LINE, and we provide support accordingly.”*

(013)

*“People from Indonesia are not familiar with winter, so we collected winter clothing together, gave them items such as boots, and taught them how to shovel snow. On snowy days, when it would be difficult for them to walk to the hospital, administrative staff would even go to pick them up.”*

(005)

In the subcategory [Supporting the needs of accompanying family members], participants <assisted migrant nurses with applications for childcare and elementary schools> and <found opportunities for their families to engage with the local community>.

*“As the migrant nurse’s child was close in age to my own, I invited them to join a baseball team together. I hoped that, by getting to know people not only in the hospital but also in the local community, they would be able to make friends more quickly. In this way, support for living in the community was informally provided by everyone (Japanese staff) as an extension of their daily lives.”*

(015)

#### 3.2.2. Support Needs of Japanese Nurses Working Alongside Migrant Nurses (Table 3)

The support needs of Japanese nurses were classified into three main categories: “Learning about nursing practices and cultural backgrounds of migrant nurses,” “Information exchange on how to support migrant nurses,” and “Support systems for enhancing migrant nurses’ competencies in Japan”.

- Learning about Nursing Practices and Cultural Backgrounds of Migrant Nurses

This category comprised two subcategories and four codes. In the first subcategory, [Opportunities to learn about healthcare systems and nursing practices in migrant nurses’ home countries], the participants expressed a desire to <be taught the differences between nursing practices in Japan and those in migrant nurses’ home countries, so that this knowledge could be applied to their education>.

*“In China, personal hygiene care and diaper changes are generally handled by family members, so there were some migrant nurses who didn’t do those care. I was even told, ‘That’s not my job.’ I really think that if we were told in advance, ‘Chinese nurses handle these kinds of nursing tasks,’ it would be very helpful for us as educators to understand, ‘Oh, I see—nursing tasks are different here.’”*

(001)

In the second subcategory, [Opportunities to better understand the cultural backgrounds of migrant nurses], the participants described support needs such as <I want to understand the cultural differences between migrant nurses’ home countries and Japan to better connect with them> and <I want to understand the characteristics of communication styles of migrant nurses that differ from those of Japanese people to reduce misunderstandings>. Such knowledge was perceived as essential for improving educational approaches.

*“(When migrant and Japanese nurses work together) I think cultural differences are indeed very important. (For migrant nurses) they may feel strongly that ‘things were different from their home countries,’ so if we are aware of that, it can help us be more considerate.”*

(004)

*“In the Philippines, when people ask, ‘What did you just say?’, they say, ‘Huh?’ When Japanese people hear that, they tend to find that rude. Whether it’s gestures or words… Japanese nurses need to understand that foreigners aren’t necessarily doing or saying things with bad intentions.”*

(002)

- Information Exchange on How to Support Migrant Nurses

This category comprised two subcategories, [Opportunities to learn about the specific support needs of migrant nurses working in Japan] and [Opportunities to share experiences and strategies with Japanese nurses supporting migrant nurses in other hospitals], and four codes. Although participants had already made various efforts to support migrant nurses, they wished to strengthen their support by gaining a deeper understanding of migrant nurses’ needs. Specifically, the participants expressed a need to <understand the challenges faced by migrant nurses> and <exchange concerns and practical strategies to support migrant nurses with peers in other institutions>.

*“I think it would be easier for us (Japanese nurses) to support migrant nurses if we understood the challenges they face while working in Japan.”*

(011)

*“I couldn’t find much information on how other hospitals actually support migrant nurses, and I really wanted to know more about that… I often found myself wondering, ‘Are we the only ones struggling this much?’.”*

(005)

- Support Systems for Enhancing Migrant Nurses’ Competencies in Japan

This category comprised three subcategories, [Provision of Japanese language training tailored to clinical nursing practice], [Development of educational resources specifically designed for migrant nurses], and [Opportunities to promote peer support among migrant nurses], and five codes. The code included the need for <practical language training focused on patient communication>, <educational materials accessible in migrant nurses’ native languages>, and <networking opportunities among migrant nurses across institutions>.

*“The biggest challenge (for migrant nurses) is communication. I think it would be helpful to have (educational) support on how to communicate with patients while working as a nurse in Japan.”*

(016)

*“If there were a system where they (migrant nurses) could access e-learning (on nursing skills) in their native language, I think it would make learning much easier. Migrant nurses come prepared to study, but often they don’t actually understand the material very well. If they could study in their native language, I think it would make it significantly easier for us (Japanese nurses) to teach them.”*

(006)

*“If nearby hospitals are hiring migrant nurses, having opportunities for them to interact might allow them to communicate with each other, sharing things like, ‘We do this at our hospital.’ I think it would be good to have opportunities like that.”*

(001)

These findings indicate a need for structured, multilevel support systems that extend beyond individual hospitals.

## 4. Discussion

### 4.1. Efforts Made by Japanese Nurses to Work Alongside Migrant Nurses

#### 4.1.1. Support to Complement Japanese Language Skills

Globally, language has been identified as one of the most significant barriers to the international mobility of nurses [16]. In nursing practices, communication skills are essential for ensuring patient safety and facilitating effective collaboration among healthcare professionals [17]. Therefore, language barriers affecting migrant nurses may increase the risk of miscommunication, potentially leading to errors and compromising patient safety [18].

Under the EPA framework, migrant nurses are required to have passed the Japanese Language Proficiency Test at the N3 level (i.e., the ability to understand Japanese used in everyday situations to a certain extent) or the N4 level (i.e., the ability to understand basic Japanese) in order to work in Japan [2,19]. However, the present findings suggest that achieving these levels of language proficiency does not necessarily translate into functional competence in clinical settings. One possible explanation is that communication in clinical settings often involves rapid interactions using specialized terminology that differs from the formal language migrant nurses learn in textbooks. Additionally, Japanese patients frequently use indirect expression, which may further increase the difficulty of communication. Even after gaining several years of experience as nursing assistants and passing the national nursing examination, migrant nurses may continue to experience difficulties in performing essential tasks, such as writing nursing records using specialized medical terminology, conducting handover briefings, and accurately understanding patients’ concerns. Consequently, the Japanese nurses utilized adaptive communication strategies, including the use of “easy Japanese” during handovers and clinical interactions. At the same time, they carefully monitored migrant nurses’ comprehension and adjusted their explanations accordingly.

“Easy Japanese” is intended to convey key information clearly and concisely by avoiding ambiguous or euphemistic expressions; however, its effective use requires specific communication skills and is not inherently intuitive, even for native Japanese speakers [20].

Although Japanese nurses developed practical tools to facilitate migrant nurses’ understanding, they perceived that migrant nurses continued to require ongoing opportunities for Japanese language learning. Previous research has similarly indicated that migrant nurses themselves seek continuous opportunities to improve their Japanese language proficiency [21]. Given the demanding and time-constrained nature of clinical practice, there are inherent limitations to the extent to which Japanese nurses can provide sustained language support within the workplace. These findings highlight the need for structured and accessible language support systems beyond individual workplace efforts. Specifically, the development of tailored Japanese language learning resources for migrant nurses, such as e-learning programs that enable flexible and independent study, is warranted. Such approaches may enhance communication competence, reduce communication-related risks, and ultimately contribute to safer patient care and more effective interprofessional collaboration in multicultural healthcare settings.

#### 4.1.2. Support for Migrant Nurses to Overcome Differences

The participants in this study provided multifaceted support to help migrant nurses address the linguistic, cultural, religious, and professional differences encountered when adapting to nursing practice in Japan. These differences extended beyond clinical tasks and reflected broader challenges associated with working in an unfamiliar healthcare system and sociocultural environment. Research from other countries has also shown that differences in operational standards and workplace culture compared with those in migrant nurses’ home countries hinder their ability to adapt [5]. Previous studies have highlighted the importance of workplace support for migrant nurses’ adaptation. However, less attention has been paid to the specific ways in which host-country nurses facilitate this process in everyday clinical practice. The novelty of this study lies in demonstrating that, through these interactions, Japanese nurses developed an understanding of the lived experiences of migrant nurses working far from their home countries and functioned as key individuals who supported their adaptation and fostered their self-confidence in daily practice. Such support is crucial because failure to recognize migrant nurses’ authority and expertise may lead to deskilling and perceived disempowerment [22]. Participants in nursing management positions more frequently emphasized recognizing migrant nurses’ existing competencies to assign roles that leveraged their prior experiences and strengths.

- Religion

In line with previous studies [11,23], this study indicates that religious considerations in the workplace are essential owing to the differing sociocultural backgrounds of migrant nurses compared with Japanese staff. According to one survey report, the majority of Japanese people do not adhere to a specific religion; among those who do, approximately 31% are Buddhist, 3% are Shinto, and approximately 1% are Christian [24]. Because estimates of religious affiliation vary depending on survey definitions and methods, these figures should be interpreted as source-dependent estimates rather than definitive population values. Consequently, there may be limited familiarity with religions such as Islam and Christianity, which are commonly practiced by migrant nurses. Furthermore, as Japanese workplace culture generally tends to separate personal matters, including religion, from professional settings, staff may find it difficult to fully understand the immediate needs of migrant nurses related to dietary restrictions, prayer practices, and religious observances.

Japanese nurses not only accommodated migrant nurses’ religious practices but also raised awareness among colleagues about the importance of such accommodations. Nevertheless, given the constraints of busy clinical settings, migrant nurses may still face challenges in observing their religious practices to the same extent as in their home countries [23]. These findings suggest that institutional-level policies and workplace education on religious diversity are necessary to support consistent and equitable accommodations.

- Nursing Practices

Differences in roles and nursing practices between migrant nurses’ home countries and Japan can act as barriers to clinical adaptation [18] and may contribute to uncertainty in professional identity [25]. The findings of this study indicate that Japanese nurses played a critical role in bridging these differences by explicitly explaining the values and expectations underlying Japanese nursing practice. In particular, they supported migrant nurses who were unfamiliar with tasks such as assisting with personal care, which may not traditionally be considered part of nursing responsibilities in their home countries.

Through these efforts, Japanese nurses facilitated migrant nurses’ understanding of the purpose and meaning of patient-centered care in Japan, including the emphasis on daily living support and patient comfort. Because differences in nursing care practices and professional autonomy can affect migrant nurses’ adaptation to a new work environment, structured orientation programs for migrant nurses are essential [22]. Such support may help migrant nurses reconcile differences in professional roles and integrate these practices into their own nursing identity. A study of migrant nurses in Germany also indicated that being recognized and respected as professional nurses in the workplace facilitated their adaptation to the host country’s healthcare environment [26].

- Personal Life

The findings of this study indicate that Japanese nurses extended their support beyond the clinical setting. Such support reflects an understanding that migrant nurses often face difficulties not only in the workplace but also in adapting to life in a new sociocultural environment. Japanese nurses recognized these broader needs and supported migrant nurses’ continued employment by providing guidance on matters such as children’s education and family employment.

This finding highlights the important role of informal, relationship-based support in facilitating the social integration of migrant nurses. The comprehensive support provided by Japanese nurses, which extends into the private lives of migrant nurses, may contribute to their sense of belonging and stability, thereby encouraging them to settle in Japan and continue working [10]. However, reliance on individual goodwill may place additional burdens on Japanese nurses, potentially affecting their personal lives and undermining the sustainability of support for migrant nurses. These findings suggest the need for organizational mechanisms to support migrant nurses’ social integration more systematically.

### 4.2. Support Needs of Japanese Nurses Working Alongside Migrant Nurses

The results of this study revealed that, while Japanese nurses actively supported migrant nurses by considering cultural, religious, and practice-related differences, they also expressed a need to further deepen their understanding in these areas. Cultural differences and variations in nursing practices between the migrant nurses’ home countries and the host country may influence interpersonal relationships with host-country nurses [27]. Therefore, to facilitate effective collaboration in multicultural clinical settings, host-country nurses require structured educational opportunities to enhance intercultural competence [28]. According to Campinha-Bacote, this voluntary motivation to gain a deeper understanding of migrant nurses’ cultural backgrounds is termed “cultural desire”. This is one of the core components of cultural competence, conceptualized as an ongoing process of developing the ability to work effectively in diverse cultural contexts [29]. In this study, one Japanese nurse involved in educating migrant nurses had traveled to their home countries to learn about healthcare systems and nursing practices. However, such opportunities are not feasible for most nurses, indicating the need for accessible and context-appropriate learning resources that can be utilized within Japan. These may include workshops, online modules, and institutional training programs focusing on cultural, religious, and nursing practice differences. It is important to note that nurses from the same country do not necessarily share the same values, communication styles, religious beliefs, or approaches to care. Therefore, this knowledge should be viewed merely as only a starting point for building relationships.

Furthermore, the findings indicate that Japanese nurses seek opportunities to exchange information with peers at other institutions regarding effective strategies for supporting migrant nurses. Given that the number of migrant nurses varies across hospitals, individual institutions may have limited experience and resources, which can hinder the development of effective support practices. Establishing inter-institutional networks and platforms for knowledge sharing may enable Japanese nurses to access a broader range of experiences and develop more effective and standardized support approaches.

This study also revealed that Japanese nurses recognize the importance of peer support among migrant nurses and express a need to facilitate such opportunities. As networks among migrant nurses provide emotional support and contribute to their continued employment in Japan [21], creating opportunities for interaction through continuing education programs or regional networks may strengthen these support systems and enhance retention. Furthermore, the acceptance of migrant nurses may broaden Japanese nurses’ international perspectives and contribute to improved care of foreign patients [12]. Support that enables migrant nurses to fully utilize their competencies may promote greater recognition of their role in Japanese healthcare and facilitate their adaptation.

### 4.3. Limitations and Implications for Future Research

This study analyzed Japanese nurses’ efforts and support needs from the perspective of Japanese nurses only, which represents a key limitation. Future research should incorporate the perspectives of migrant nurses themselves to evaluate and validate the support practices identified in this study and to gain a more comprehensive understanding of their specific support needs.

Because this study relies on participants’ own accounts, it is subject to self-report bias. The findings may also have been influenced by the participants’ cultural values and the personal characteristics of the migrant nurses whom they had worked with. As participants were recruited through nursing directors and snowball sampling, and included nurses with managerial or educational responsibilities, the findings may primarily reflect the experiences of individuals who are particularly involved in, or attentive to, supporting migrant nurses. Therefore, the perspectives of bedside nurses with less direct involvement in organized support activities, as well as more ambivalent or negative experiences, may not have been fully captured. This may limit the transferability of the findings to all nurses working with migrant nurses in Japan.

Another limitation is that data on hospital size were not collected. As migrant nurses’ adaptation experiences may vary according to hospital size, the influence of this factor could not be assessed. In addition, because the findings are based on a qualitative sample, they may not be transferable across all clinical settings or populations of Japanese nurses working with migrant nurses. Further studies using larger and more diverse samples are warranted to validate and expand upon these findings.

Future efforts should focus on developing and evaluating educational and organizational interventions that promote collaboration between Japanese and migrant nurses, grounded in the support needs identified in this study. Online educational tools, such as e-learning modules, can provide standardized training for migrant nurses across Japan and for the Japanese nurses who work alongside them. Such initiatives may contribute to improving workplace integration, enhancing job satisfaction, and supporting the long-term retention of migrant nurses within the Japanese healthcare system.

## 5. Conclusions

This study aimed to clarify Japanese nurses’ efforts and support needs when working with migrant nurses in Japan. Semistructured interviews were conducted with 18 Japanese nurses, and the data were analyzed using a qualitative descriptive approach. The results identified five categories of efforts: “Overcoming language barriers,” “Respecting the nursing practices and cultural backgrounds of migrant nurses,” “Encouraging migrant nurses to gain self-confidence,” “Engaging Japanese staff in establishing support systems for migrant nurses,” and “Addressing the personal challenges faced by migrant nurses working abroad.” Regarding the support needs of Japanese nurses, three categories were identified: “Learning about nursing practices and cultural backgrounds of migrant nurses,” “Information exchange on how to support migrant nurses,” and “Support systems for enhancing migrant nurses’ competencies in Japan.” These findings indicate that Japanese nurses play a central role in supporting the adaptation and integration of migrant nurses through both clinical support and non-clinical support. However, the support extends beyond individual efforts, highlighting the need for structured educational programs, institutional support systems, and inter-organizational collaboration. Strengthening these systems may contribute to improving workplace integration, enhancing communication, and promoting the long-term retention of migrant nurses in Japan.

## Figures and Tables

**Table 1:** Characteristics of the Participants.

ID	Years of Nursing Experience	Professional Role	Number of Migrant Nurses Worked with	Years Working Alongside Migrant Nurses	Countries of Origin of Migrant Nurses Worked with
001	11	Educator	Three or more	4–5 years	Indonesia, Philippines, Vietnam, China
002	8	Educator	Three or more	2–3 years	Indonesia, Philippines, Vietnam
003	27	Educator	Three or more	More than 5 years	Philippines, Vietnam, China
004	12	Colleague	Two	2–3 years	Philippines
005	27	Educator	Three or more	More than 5 years	Indonesia, Vietnam
006	20	Educator	Three or more	More than 5 years	Indonesia, China
007	2	Colleague	Three or more	2–3 years	China
008	29	Educator	Three or more	4–5 years	Indonesia, Philippines
009	16	Nursing Administrator and Educator	Two	More than 5 years	China
010	25	Educator	Three or more	4–5 years	Indonesia, Philippines, Vietnam
011	6	Colleague	Two	Less than 1 year	Philippines
012	20	Educator	Three or more	2–3 years	Indonesia, Philippines
013	21	Nursing Administrator and Educator	Three or more	2–3 years	Indonesia, Philippines
014	31	Nursing Administrator	Two	1 year	China
015	30	Nursing Administrator, and Educator	One	More than 5 years	Philippines
016	4	Colleague	Three or more	2–3 years	China
017	23	Educator	Three or more	More than 5 years	Indonesia, Philippines
018	18	Colleague	Three or more	More than 5 years	Indonesia, Philippines, Vietnam, China

**Table 2:** Efforts Made by Japanese Nurses to Work alongside Migrant Nurses (N = 18).

Category	Subcategory
Overcoming language barriers	Using clear and simplified language to facilitate understanding among migrant nurses
	Providing repeated and sustained explanations until understanding is achieved
	Supporting tasks requiring advanced Japanese language proficiency
	Developing and utilizing tools to overcome language barriers
	Providing continuous opportunities for migrant nurses to learn and improve Japanese language skills
Respecting the nursing practices and cultural backgrounds of migrant nurses	Teaching Japanese nursing practices while acknowledging perspectives from migrant nurses
	Adapting instructional approaches to be culturally appropriate
	Creating work environments that accommodate religious practices
Encouraging migrant nurses to gain self-confidence	Recognizing migrant nurses as qualified professionals in their home countries
	Assigning tasks that leverage migrant nurses’ individual strengths
Engaging Japanese staff in establishing support systems for migrant nurses	Raising awareness among Japanese staff regarding the characteristics and needs of Migrant nurses
	Acting as intermediaries between migrant nurses and Japanese staff
Addressing the personal challenges faced by migrant nurses working abroad	Responding to migrant nurses’ needs related to returning to their home countries
	Providing support for challenges encountered in daily life in Japan
	Supporting the needs of accompanying family members

**Table 3:** Support Needs of Japanese Nurses Working alongside Migrant Nurses (N = 18).

Category	Subcategory
Learning about nursing practices and cultural backgrounds of migrant nurses	Opportunities to learn about healthcare systems and nursing practices in migrant nurses’ home countries
	Opportunities to better understand the cultural backgrounds of migrant nurses
Information exchange on how to support migrant nurses	Opportunities to learn about the specific support needs of migrant nurses working in Japan
	Opportunities to share experiences and strategies with Japanese nurses supporting migrant nurses in other hospitals
Support systems for enhancing migrant nurses’ competencies in Japan	Provision of Japanese language training tailored to clinical nursing practice
	Development of educational resources specifically designed for migrant nurses
	Opportunities to promote peer support among migrant nurses

## Data Availability

The data collected in this study are not available to the public for reasons of informed consent and confidentiality but may be obtained from the corresponding author upon reasonable request.

## Public Involvement Statement

No public involvement in any aspect of this research.

## Guidelines and Standards Statement

This manuscript was drafted against the SRQR [Standards for Reporting Qualitative Research] reporting guideline for writing qualitative research articles.

## Abbreviations

The following abbreviations are used in this manuscript:

EPA	Economic Partnership Agreement
